# Tropical diabetic hand syndrome: a case report

**DOI:** 10.1186/s13104-017-2405-3

**Published:** 2017-02-13

**Authors:** Eugene Vernyuy Yeika, Jacques Cabral Tchoumi Tantchou, Joyce Bei Foryoung, Paul Nkemtendong Tolefac, Derrick Tembi Efie, Siméon Pierre Choukem

**Affiliations:** 1 Saint Elizabeth Catholic General Hospital and Cardiac Centre Shisong, Kumbo, Cameroon; 2 Clinical Research Education Networking and Consultancy, Douala, Cameroon; 3 Health and Human Development Research Group, Douala, Cameroon; 4 Faculty of Medicine and Biomedical Sciences, University of Yaounde I, Yaounde, Cameroon; 5 Banso Baptist Hospital, Kumbo, Cameroon; 6 Department of Internal Medicine and Paediatrics, Faculty of Health Sciences, University of Buea, Buea, Cameroon

**Keywords:** Tropical diabetic hand syndrome, Case report, Late diagnosis, Classification

## Abstract

**Background:**

Tropical diabetic hand syndrome describes a complex hand sepsis affecting patients with diabetes across the tropics and often results from a trivial hand trauma. The clinical presentation of this syndrome is variable and ranges from localised swelling and cellulitis, with or without ulceration of the hand to progressive fulminant hand sepsis, and gangrene affecting the entire limb which may be fatal. Tropical diabetic hand syndrome could lead to permanent disability and death as a result of delay in presentation, late diagnosis and late medical and surgical intervention. This indexed case acts as an eye opener for physicians to the existence of this hand sepsis.

**Case presentation:**

We report the case of a 57 year-old black African female diabetic who was referred to our centre for the management of a suppurating ulcer and swelling of the left hand of two weeks duration. On examination and work-up, the patient was found to have Lawal Group III left diabetic hand syndrome and was managed with parenteral antibiotics, radical debridement and the hand was eventually amputated. She died 7 days following amputation from overwhelming sepsis.

**Conclusion:**

Though tropical diabetic hand syndrome is a relatively rare complication of diabetes, it can be fatal as in this case report. Early diagnosis and proper management would yield better outcome. Initial management should include aggressive intravenous broad-spectrum antibiotics with anaerobic coverage. Classification of tropical diabetic hand syndrome will assist physicians and surgeons in decision making, proper management and easy communication.

## Background

Hand complications of diabetes mellitus are rare compared to foot complications occurring in a ratio of 1:20 [1]. Tropical diabetic hand syndrome (TDHS) describes an acute complex hand complication affecting patients with diabetes in the tropics usually following a minor injury to the hand [2]. The clinical presentation of TDHS is variable and ranges from localised swelling and cellulitis, with or without ulceration of the hand to progressive fulminant hand sepsis, and gangrene affecting the entire limb which may be fatal [3]. TDHS occurs primarily in diabetic patients who live in the tropical or coastal areas [3]. The outcome of TDHS may range from limb deformity to amputation and even death [4]. Independent risk factors for TDHS include poorly controlled diabetes, peripheral neuropathy, female sex, insect bites, hand trauma, low socioeconomic status, residence in coastal areas and late presentation to the hospital [5]. TDHS is not generally classified amongst specific diabetic complications and its occurrence is often under-reported, and consequently not known to many physicians [3, 6]. This indexed case acts as an eye opener for physicians to the existence of this fatal hand sepsis.

## Patient information

A 57 year-old black African female farmer was referred to our centre for the management of a suppurating ulcer and swelling of the left hand of two weeks duration. This started following a trivial needle prick on the left palm during laundry which became swollen and pustular after one week. It was associated with pains, throbbing in nature and relieved by hand elevation. The hand was incised at home resulting in a suppurating ulcer on the palmer surface that increased in size to involve the dorsum and forearm. The patient initially sought medical attention from a traditional practitioner who managed the lesion as whitlow. She later presented at a health centre and was referred to Saint Elizabeth Catholic General Hospital Shisong.

She had a 7 year history of type 2 diabetes mellitus and was on metformin and glibenclamide but was non-compliant to treatment. She did not smoke cigarette or drink alcoholic beverages. She had no history of hypertension or impaired renal function. She reported loss of sight of the left eye following an eye surgery indicated for correction of striate keratopathy and glaucoma 2 years prior to presentation and numbness of the extremities of 6 months duration.

**Fig. 1:**
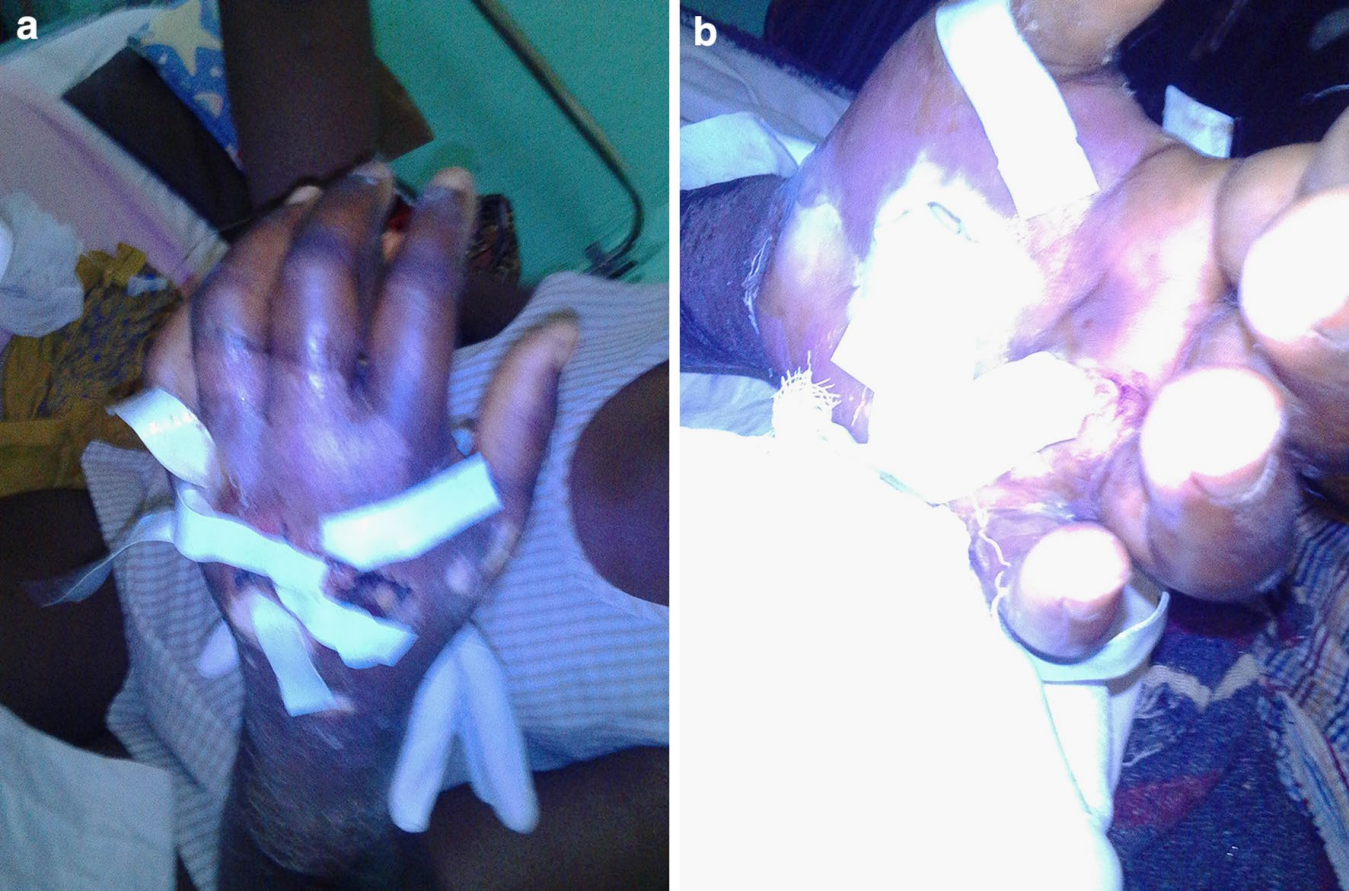
Left hand showing ulcers and suppurations. **a** Dorsal view. **b** Palmar view

Physical examination revealed an ill-looking woman, febrile (temperature = 38.3 °C) with pale conjunctivae. Her left hand was swollen, warm, fluctuant and ulcerated with foul smelling copious discharge. There was progressive cellulitis and wet gangrene up to the mid forearm (Fig. 1). Sensations were reduced on both extremities.

Investigations conducted included a random blood glucose of 33.3 mmol/l (measured upon admission), urine analysis that showed no ketone bodies, full blood count that revealed a leukocytosis of 15.5 × 10^3^/µl with neutrophil predominance of 12.4 × 10^3^/µl (81%) and moderate microcytic hypochromic anaemia with hemoglobin level of 7.8 g/dl. Serum electrolytes were within their normal ranges, blood urea nitrogen was 102.8 mg/dl and creatinine was 5.0 mg/dl giving a urea/ creatinine ratio of 20.6. A wound swab and culture isolated *Enterobacter* spp. which was sensitive to ciprofloxacin, gentamycin, ceftriaxone and chloramphenicol.

A diagnosis of Lawal Group III left diabetic hand syndrome was made.

After counselling and consent, aggressive surgical debridement was done under local anaesthesia. Other modalities of management included parenteral antibiotics (ceftriaxone 1 g 12 h and metronidazole 500 mg 8 h), tight glycaemic control (using sliding-scale soluble insulin), analgesics (parenteral paracetamol 1 g 8 h) and fluids. The wet gangrene of the hand and forearm progressed despite aggressive antibiotics, repeated debridement and drainage. The patients’ condition worsened by the installation of septic shock that was addressed with timely intravenous hydrocortisone (100 mg IV bolus) and epinephrine (1 mg IV bolus). After resuscitation, the left hand was amputated. Postoperative evolution was marked by swinging pyrexia and alteration in the level of consciousness and blood culture was requested. She eventually went into coma and died on the 7th day following surgery from overwhelming sepsis.

## Discussion

Hand infections and ulceration remain a major cause of morbidity and mortality amongst diabetics in the tropics [4, 7]. Abbas et al. in 2001 reported a mortality rate of 13% from overwhelming sepsis due to TDHS [5]. We report this case to highlight the factors that increase disability or death from TDHS and the necessity to adopt a common classification for TDHS to guide physicians in decision making and ease communication.

Delay in presentation at the hospital and late initiation of medical treatment are common in patients with TDHS [8]. Such delays occur because of the low socioeconomic status, limited access to medical care or unawareness of the potential risks involved in hand infections amongst patients with diabetes [8, 9]. Our patient did not benefit from early and complete broad spectrum antibiotics partly because she presented late to the hospital and because of her poor socioeconomic status as she could not purchase drugs on time. Previous reports show that these patients often underestimate the gravity of TDHS as the initiating trauma is often minor [8–10]. Due to ignorance and poverty, they first tend to home treatment or seek initial help from traditional healers [6, 8, 9].

Since the first report of TDHS in Africa in 1984 [11], its occurrence has been under-reported [9] and consequently very few physicians know about its existence [6, 12]. Lack of knowledge on TDHS results in late diagnosis and late initiation of proper management upon presentation. TDHS is commoner amongst women, as women in the tropics are often engaged in household chores and farming which predispose them to hand trauma [9].

Routine wound swabs in TDHS do not guide optimal antimicrobial therapy and should be avoided in hospitals with limited laboratory facilities [9]. According to Gill et al. culture of tissue biopsy specimens yields single bacterial species in over 75% whereas swab cultures yields polymicrobial flora in the majority of cases probably due to contamination [13]. Relying on culture results before initiation of broad-spectrum antibiotics delays management and contributes to development of septicemia, septic shock and eventual death.

Unlike diabetic foot infections, no classification has been adopted for TDHS despite the rising need for a classification that will enable early decision making, proper management and good communication amongst physicians [1]. Lawal et al. in 2013 described a new course of management based on a proposed new classification of TDHS [1]. They classified TDHS into three groups (I–III) based on the increasing order of severity of the disease and prognosis. This recent classification by Lawal and colleagues has not yet been adopted and used and is limited as it does not relate a group to a medical or surgical intervention. Our patient had digital and hand gangrene which was group III according to Lawal classification and optimal management of this class should include debridement and/or amputation.

## Conclusion

Though tropical diabetic hand syndrome is a relatively rare complication of diabetes, it can be fatal as in this case report. Early diagnosis and proper management yields better outcomes. Initial management should include aggressive intravenous broad-spectrum antibiotics with anaerobic coverage. Classification of tropical diabetic hand syndrome will assist physicians and surgeons in decision making, proper management and ease communication.

## Abbreviation

TDHS: tropical diabetic hand syndrome
